# Profile of Urinary Cytokines in Kawasaki Disease: Non-Invasive Markers

**DOI:** 10.3390/diagnostics11101857

**Published:** 2021-10-08

**Authors:** Hsin-Chun Huang, Ho-Chang Kuo, Hong-Ren Yu, Hui-Chen Huang, Jen-Chieh Chang, I-Chun Lin, I-Lun Chen

**Affiliations:** 1 Department of Pediatrics, Kaohsiung Chang Gung Memorial Hospital and Chang Gung University College of Medicine, Kaohsiung 833, Taiwan; drhhuang@hotmail.com (H.-C.H.); erickuo48@yahoo.com.tw (H.-C.K.); yuu2002@cgmh.org.tw (H.-R.Y.); flower_04@yahoo.com.tw (H.-C.H.); rj_chang@yahoo.com.tw (J.-C.C.); mslinichun@gmail.com (I.-C.L.); 2 School of Traditional Chinese Medicine, College of Medicine, Chang Gung University, Linkou 333, Taiwan; 3 Kawasaki Disease Center, Kaohsiung Chang Gung Memorial Hospital, Kaohsiung 833, Taiwan

**Keywords:** coronary artery, cytokine, fever, Kawasaki disease, urine

## Abstract

This cohort study aimed to investigate urinary cytokines expression to help identify a less invasive method of cytokine detection for Kawasaki disease (KD). Patients with confirmed KD were recruited. Patients with fever or urinary tract infection (UTI) were enrolled as control groups. Urinary samples were collected before and 3 days after intravenous immunoglobulin (IVIG) treatment. The levels of cytokines were detected by MILLPLEX^®^ MAP human multiplex assay. All cytokines, i.e., epidermal growth factor (EGF), interferon (IFN)-γ, interleukin (IL)-1β, IL-2, IL-4, IL-5, IL-6, IL-8, IL-9, IL-10, IL-13, IL-17A, IL-33, interferon-gamma-induced protein (IP)-10, macrophage inflammatory protein (MIP)-1β, tumor necrosis factor (TNF)-α, and vascular endothelial growth factor (VEGF) except monocyte chemoattractant protein (MCP)-1 were significantly higher in the KD group, compared with the fever-control (FC) group, whereas the expressions of IFN-γ, IL-1β, IL-6, IL-8, IL-17A, IL-33, MCP-1, MIP-1β, and TNF-α were significantly lower in the urine of KD patients, as compared with the UTI group. The expressions of EGF, IFN-γ, IL-8, IL-13, and IL-17A were higher in the urine of KD patients than in the FC group, whereas the level of IL-1β was lower in KD than in the UTI group after age adjustment by logistic regression. Levels of IL-6, IL-8, IL-13, IP-10, and MCP-1 were significantly higher in the pre-IVIG urine of KD patients than in the post-IVIG treatment group. Additionally, urine IL-4 and blood C-reactive protein were higher in the KD group with coronary artery lesion (CAL) than in the non-CAL group. Results of this study provide a new view of urinary cytokine expression in the disease progress of KD, which may help clinicians to predict and prevent morbidity early and non-invasively.

## 1. Introduction

Kawasaki disease (KD) is an acute vasculitis syndrome with unknown etiology that affects multiple organ systems and occurs primarily in children younger than the age of 5. It was first reported in the English literature in 1974 as “a new infantile acute febrile mucocutaneous lymph node syndrome” [1]. KD is characterized by prolonged fever, non-suppurative lymphadenopathy, diffuse mucosal inflammation, conjunctivitis, indurative edema of the hands and feet associated with peeling of fingertips, and polymorphous skin rashes. The most severe complication of KD is the development of coronary artery lesions (CAL) [2]. Sequelae of vasculitis with coronary artery aneurysms were shown to develop in 20–25% of untreated children [3]. A multicenter study in the United States found that a single high dose of 2 g/kg intravenous immunoglobulin (IVIG) plus aspirin was able to lower the incidence of aneurysms to 3–5% [4]. Many studies have either failed to identify a pathogen responsible for KD, or the identified pathogen was not identical between studies from different laboratories. This suggests that the genetic background may be more important than the infectious pathogen contributing to KD susceptibility. The current hypothesis is that genetically susceptible children are exposed to an unknown trigger that elicits an immune response directed against components of the arterial wall [5].

Aside from the involvement of the skin, mucosa, and heart, sterile pyuria, proteinuria, and microscopic hematuria are also noted in KD and are associated with disease severity [6]. The diagnosis of febrile urinary tract infection (UTI) in children and infants requires finding both pyuria and a single uropathogenic organism consistent with UTI [7]. Extensive research on the blood and cardiovascular system in both humans and animals, as well as on some of the urinary cytokines involved in KD, has been conducted. For example, several previous studies showed the involvement of different cytokines in KD patients’ blood specimens [8,9,10]. However, although studies were conducted on urine cytokines expression in KD, only a few cytokines were actually studied [11,12,13]. Given the lack of data on various cytokines in the KD treatment process, this study aimed to search for a less invasive method of cytokine detection and to explore the roles of T_H_1, T_H_2, T_H_17, and Treg cytokines from KD patients’ urine in the regulation of inflammatory reaction in KD.

## 2. Materials and Methods

### 2.1. Patient Enrollment

Patients admitted to Chang Gung Memorial Hospital, Kaohsiung, Taiwan, who met the diagnostic criteria for KD were included. During the same period, febrile patients who were hospitalized were recruited as a control group. After admission, age, body weight (BW), lab examinations, including complete blood counts, C-reactive protein (CRP), blood urea nitrogen (BUN), creatinine (Cr), urine–creatinine (U–Cr), urine routine and sediment (UA), and culture were performed. The control group was further divided into fever and urinary tract infection (UTI) subgroups. Fever-control (FC) patients were hospitalized because of viral or bacterial infection (e.g., herpangina, pharyngitis, tonsillitis, sinusitis, cellulitis, bronchiolitis, bronchopneumonia, gastroenteritis, enterocolitis, and infectious mononucleosis). Patients with UTI were confirmed as pyuria with a single uropathogenic organism consistent with UTI diagnosis [7]. Blood and urine samples were obtained at admission for the control group, before IVIG treatment (pre), and 3 days after IVIG treatment (post-IVIG) for the KD group. They received a single infusion of IVIG (2 g/kg) administered over a 12 h period. Aspirin (3–5 mg/kg/day) was administered until all signs of inflammation were resolved or regression of coronary artery lesion (CAL), the most serious complication of KD, which may include myocardial infarction or coronary artery aneurysms, was observed. This was detected using two-dimensional echocardiography, and these conditions were the same as those in our previous study [9]. KD patients with coronary artery ectasia or dilatation that disappeared within the initial 2 months after the onset of illness were defined as transient ectasia (or transient CAL) and not judged as CAL. CAL was defined by the internal diameter of the coronary artery being at least 3 mm (4 mm, if the subject was older than age 5 years) or the internal diameter of a segment being at least 1.5 times that of an adjacent segment, as observed in the echocardiogram [9].

### 2.2. Measurement of Urinary Cytokines

After centrifugation of 0.5 mL of obtained urine for 10 min at 1000× *g*, the supernatants were collected and stored at −20 °C until batch analysis. Levels of urinary epidermal growth factor (EGF), interferon (IFN)-γ, interleukin (IL)-1β, IL-2, IL-4, IL-5, IL-6, IL-8, IL-9, IL-10, IL-13, IL-17A, IL-33, interferon-gamma-induced protein (IP)-10, monocyte chemoattractant protein (MCP)-1, macrophage inflammatory protein (MIP)-1β, tumor necrosis factor (TNF)-α, vascular endothelial growth factor (VEGF), and creatinine were examined using MILLIPLEX^®^ MAP human cytokine/chemokine magnetic bead panel assay (EMD-Millipore; Billerica, MA, USA) according to the manufacturer’s instructions. In brief, 25 μL of urinary samples were mixed with multiplexed antibody-conjugated beads before being subjected to multi-channel detection of the bead array. Each kit includes a low- and high-quality control system to ensure assay performance. A Luminex MAGPIX_TM_ (Luminex Corporation, Austin, TX, USA) instrument was used to run plates and generate quantitative data and that were acquired using Luminex^®^ xPONENT software and analyzed using MILLIPLEX^®^ Analyst 5.1 software (EMD-Millipore). The calibration of cytokine concentrations was determined by interpolation of a series of standard samples following the manufacturer’s recommendation [14,15].

### 2.3. Statistical Analysis

The data were compared by Wilcoxon matched pairs test for the pre- and post-IVIG treatment pairs of KD. Urine cytokine data were analyzed by the Mann–Whitney *U* test or the Kruskal–Wallis test, followed by Dunn post hoc tests when appropriate. The chi-squared test or Fisher’s exact test was used for the analysis of categorical variables. Continuous variables are expressed as the median and interquartile range (IQR). Categorical variables are presented as numbers. Adjusting for age by logistic regression analyses were performed to ascertain the most significant influences for KD and the effects were calculated as odds ratio (OR) and 95% confidence interval (CI). All data were analyzed statistically using IBM SPSS 25 statistics software (IBM Corp., Armonk, NY, USA), and *p* < 0.05 was considered statistically significant. Graphic assessment of cytokines was obtained by GraphPadPrism 8 (GraphPad Software, San Diego, CA, USA) and R 4.0.4 (R Foundation for Statistical Computing, Vienna, Austria).

## 3. Results

### 3.1. Demographic and Clinical Data of Included Patients

A total of 37 patients with confirmed KD were enrolled in this study, and 70 control patients with fever during the same period were included as controls. Among these 70 patients, 28 were of UTI. The median (IQR) age of FC, KD and UTI groups were 40.59 (13.50, 60.25), 23.25 (8.50, 26.50), and 3.67 (1.68, 3.90) months, (*p* < 0.01 for KD/UTI). The median BW of FC, KD and UTI groups were 15.87 (10.63, 7.73), 12.07 (8.65, 11.45), and 6.02 (5.07, 6.90) kg, (*p* < 0.01 for KD/UTI, *p* < 0.05 for KD/FC). In addition, significant differences were found in hemoglobin, hematocrit, platelet, CRP, urinary WBC (U-WBC), and U-creatinine (Cr) between KD and FC groups, as well as in CRP, urinary RBC (U-RBC), and U-Cr between KD and UTI groups, as shown in Table 1. KD patients with CAL had significantly higher CRP levels than non-CAL patients (Table 1).

### 3.2. Cytokines Expression in Urine

**Figure 1:**
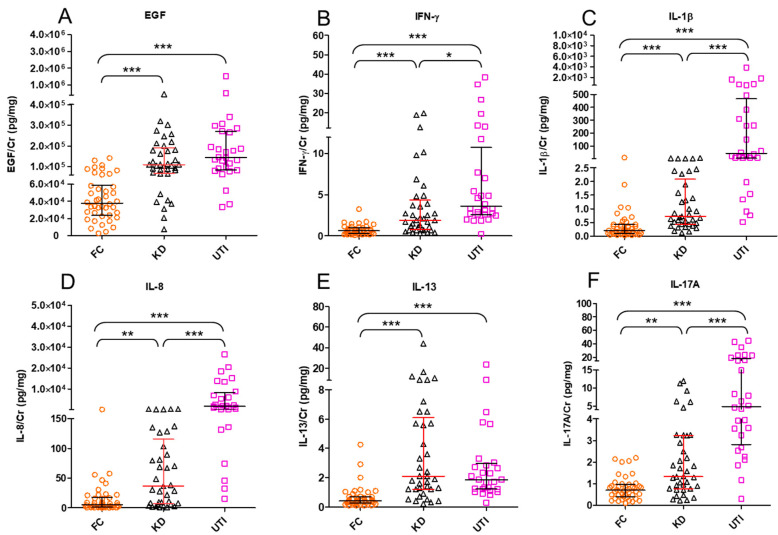
Differences in urinary cytokines between fever-control (FC), Kawasaki disease (KD), and urinary tract infection (UTI) groups. Levels of urinary cytokines corrected to urinary creatinine (Cr) in (**A**) epidermal growth factor (EGF), (**B**) interferon (IFN)-γ, (**C**) interleukin (IL)-1β, (**D**) IL-8, (**E**) IL-13, and (**F**) IL-17A are expressed as median (IQR) by Kruskal–Wallis test. Asterisk indicates statistical significance * for *p* < 0.05, ** for *p* < 0.01, and *** for *p* < 0.001.

**Figure 2:**
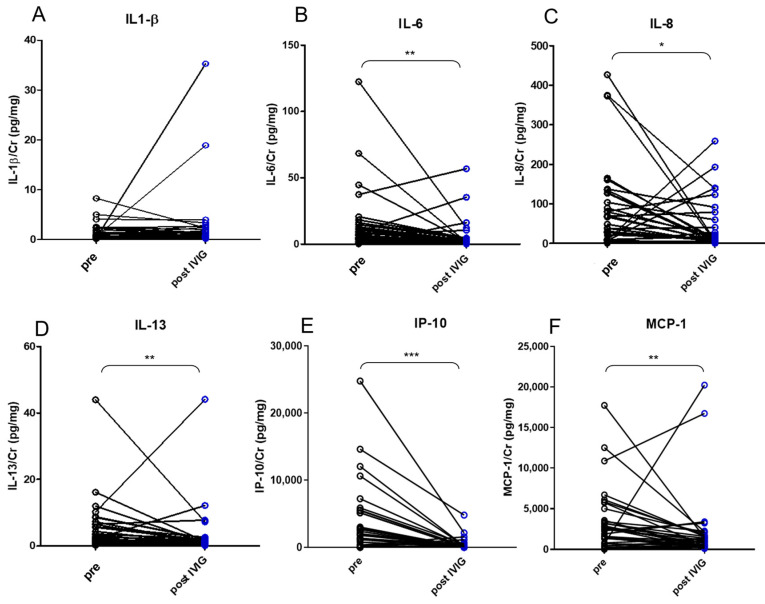
Changes in urine cytokines corrected to urine creatinine: (**A**) interleukin (IL)-1β, (**B**) IL-6, (**C**) IL-8, (**D**) IL-13, (**E**) interferon-gamma-induced protein (IP)-10, and (**F**) monocyte chemoattractant protein (MCP)-1 from patients with Kawasaki disease before (pre) and three days after (post) intravenous immunoglobulin (IVIG) treatment, tested by Wilcoxon matched pairs test. * for *p* < 0.05, ** for *p* < 0.01 and *** for *p* < 0.001.

**Figure 3:**
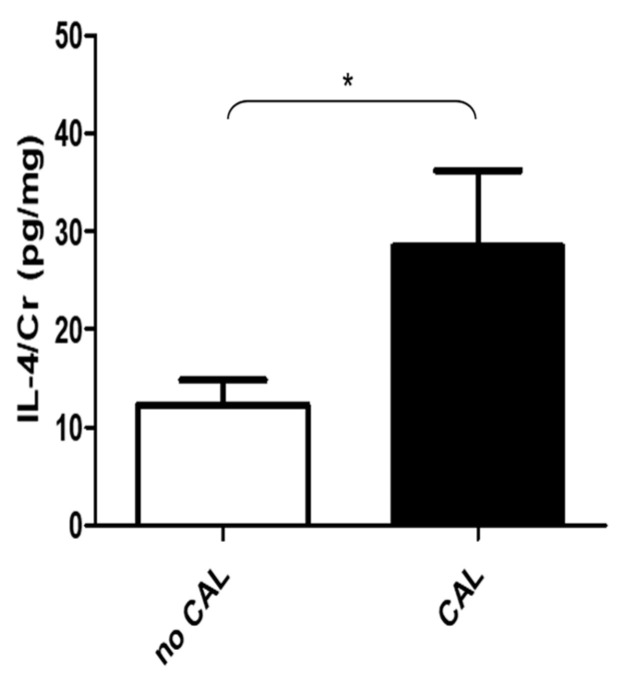
Urinary IL-4/urinary creatinine from KD patients with or without coronary artery lesion (CAL), tested by Mann–Whitney *U* test. * for *p* < 0.05.

**Figure 4:**
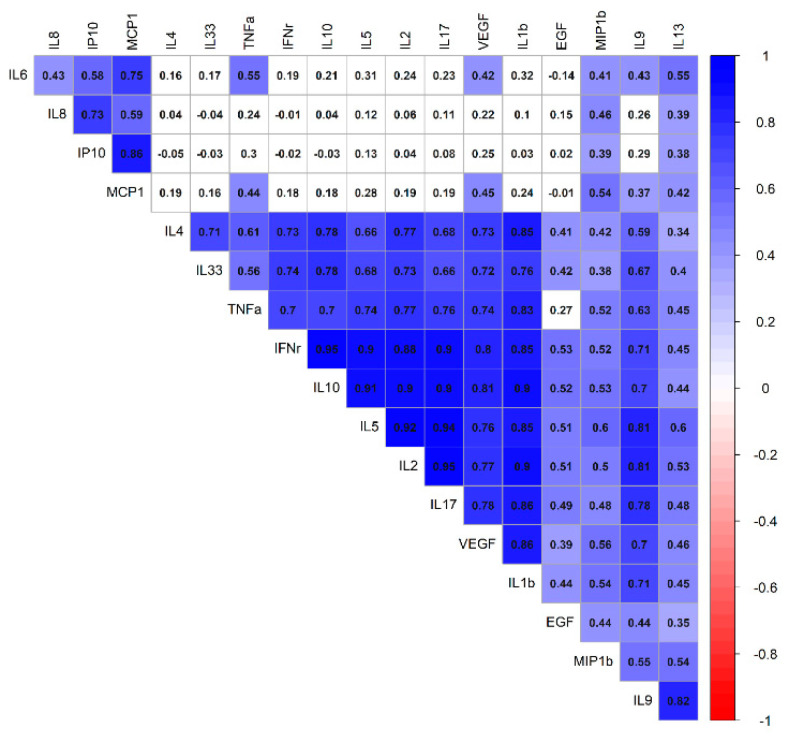
The correlation of various urine cytokines in Kawasaki disease before treatment. In the correlation matrix, the Spearman correlation coefficients are used (blue for positive correlations and red for negative correlations).

Urine specimens of all enrolled patients were collected during hospitalization. In KD patients, urine was collected twice, before and after 3 days of IVIG treatment. All measured urinary cytokines: EGF, IFN-γ, IL-1β, IL-2, IL-4, IL-5, IL-6, IL-8, IL-9, IL-10, IL-13, IL-17A, IL-33, IP-10, MCP-1, MIP-1β, TNF-α, and VEGF were corrected on the basis of urinary creatinine concentrations, expressed as picograms of urine cytokine per milligram of urine creatinine (pg/mg). All cytokines, i.e., EGF, IFN-γ, IL-1β, IL-2, IL-4, IL-5, IL-6, IL-8, IL-9, IL-10, IL-13, IL-17A, IL-33, IP-10, IFN-γ, MIP-1β, TNF-α, and VEGF except MCP-1 were significantly higher in the KD group, compared with the FC group, whereas the expression of IFN-γ, IL-1β, IL-6, IL-8, IL-17A, IL-33, MCP-1, MIP-1β, and TNF-α were significantly lower in the urine of KD patients, as compared with the UTI group, as tested by the Kruskal–Wallis test (Table 2). After adjusting for age by the logistic regression model, the levels of EGF, IFN-γ, IL-8, IL-13, and IL-17A were significantly higher in the urine of KD than in the FC group. In addition, the expression of IL-1β was found significantly higher in the urine of UTI patients, as compared with the KD group after adjusting for age (Table 3 and Figure 1). Furthermore, the comparison between before and three days after IVIG treatment showed that the expression of IL-6, IL-8, IL-13, IP-10, and MCP-1 were significantly higher in the urine of KD patients before treatment, tested by Wilcoxon matched pairs test (Figure 2). In addition, urinary IL-4 from Kawasaki disease patients with or without coronary artery lesion (CAL) were compared by Mann–Whitney *U* test and showed IL-4 was significantly higher in the CAL group (Figure 3). The correlation of various urinary cytokines in KD before treatment was analyzed by Spearman’s rank correlation test and shown in Figure 4.

## 4. Discussion

Results of the present study revealed that the expressions of IL-6, IL-8, IL-13, IP-10, and MCP-1 were significantly higher in the urine of KD patients before treatment, compared with three days post-IVIG treatment. Moreover, urine IL-4 and blood CRP levels were higher in the CAL than in the non-CAL groups. Most urinary cytokines were significantly higher in KD, compared with the FC group (Table 2). In addition, expressions of cytokines EGF, IFN-γ, IL-8, IL-13, and IL-17A were higher in the urine of the KD group than the FC group after adjusting for age, whereas the expression of IL-1β was significantly higher in the urine of UTI, as compared with the KD group after age adjustment (Table 3). This is the first study to demonstrate profiles of various urinary cytokines in KD patients’ urine.

Our previous studies and others had shown that IL-6, IL-8, IL-10, IL-17A, IP-10, MCP-1, and TNF-α were elevated in plasma from KD patients in the acute phase and would decline significantly after IVIG treatment [9,10,16,17,18]. Most of these cytokines were in parallel to the expression of urinary cytokines from KD patients in the acute phase and declined after IVIG treatment. However, plasma IL-2, IL-4, and IL-5 increased significantly post-IVIG treatment [16,19], but changes in the urine from KD patients were not significant. The present study found that urinary IL-6, IL-8, IL-13, IP-10, and MCP-1 decreased significantly post-IVIG treatment, and the most significant decrease was in IP-10.

IP-10 is a potent chemoattractant of small-molecular-weight (10 kDa). Additionally, known as C-X-C motif chemokine ligand 10 (CXCL10), which is secreted by monocytes, neutrophils, eosinophils, epithelial, endothelial, and stromal cells, and keratinocytes in response to IFN-γ in inflammation, it promotes migration of monocytes and activated T cells to inflammation foci [20,21]. IFN-γ, a T_H_1 cytokine secreted mostly by natural killer cells and CD8+ T cells, can recruit macrophages and T cells into coronary arteries [22]. Serum levels of IFN-γ were reported to be increased in KD, compared with controls [23,24], and stimulated mononuclear cells from KD patients can produce significantly higher levels of IFN-γ [25]. Our data of IFN-γ were higher in urine from KD, compared with FC but not the UTI group after age adjustment. IP-10 is a downstream mediator of IFN-γ. Our previous study demonstrated that plasma IP-10 is a biomarker for KD [10], and its genetic polymorphisms were highly associated with high-risk KD [26]. In the present study, urinary IP-10 levels declined more significantly in KD patients after 3 days of IVIG treatment compared to urinary IL-6. Other investigators had shown that urinary IL-6 returned more rapidly to the baseline level than urinary levels of N-acetyl-β-D-glucosaminidase (NAG) and β_2_-microglobulin (β_2_-mg), the parameters of renal tubular damage, and serum IL-6 [13]. Furthermore, increases in urinary IL-6 correlated with elevated renal Doppler measures, implying renal vasculitis in KD [12]. Plasma proinflammatory cytokines (e.g., IL-1β, TNF-α, IL-8) have been shown to have a low molecular weight (<20 kd) and underwent glomerular filtration, followed by denaturation by the proximal tubular cells [27,28]. By contrast, anti-inflammatory cytokine IL-10 is of greater molecular weight than proinflammatory cytokines (>20 kd) and thus is less readily filtered by the glomerulus [29]. Thus, it was postulated that immune-mediated renal vasculitis and renal tubular damage developed in the acute phase of KD, revealing the improvement after IVIG and aspirin treatment.

CAL is a serious complication of KD that may remain with children even as they progress into adulthood [30,31]. It occurs in 20–25% of untreated children [3], which reduces to 3–5% with IVIG plus aspirin treatment [4,32]. Many studies have found biomarkers in human [33,34] and animal models [35]. The present study describes, for the first time, that urine IL-4 levels were higher in KD with CAL than non-CAL before IVIG treatment. This was in line with the results of a study demonstrating that serum IL-4 levels in KD patients with CAL were higher than those without CAL [36], which suggests that the IL-4 either in serum or urine may be associated with CAL. IL-4 is a cytokine of 17 kDa molecular weight that induces differentiation of naive helper T cells (T_H_0 cells) to T_H_2 cells, secreted primarily by mast cells, T_H_2 cells, eosinophils, basophils, and modulates cytokine production by endothelial cells and monocytes [37,38]. IL-4 may induce the expression of a cell surface VCAM-1-like adhesion molecule, which causes an increase in lymphocyte, eosinophil, and basophil adhesion to the endothelium, resulting in transendothelial migration to local inflammatory sites [39].

Unlike IL-1α, which is both constitutively expressed and active in its 31 kDa pro-form, IL-1β is only formed in its inactive 35 kDa pro-form following priming signals, such as pathogen- or damage-associated molecular patterns, and is only later cleaved to its 17 kDa active form following inflammasome activation in a damaged or diseased state. Reduction of IL-1β secretion in KD patients post-IVIG treatment has been noted [40,41]. IVIG was also shown to downregulate IL-1 and upregulate IL-1Ra production in vitro [42,43]. Several clinical studies on KD suggest that serum levels of IL-1β or IL-1β gene expression may play an essential role in KD [8,44]. IL-1β expression increased with age in a young mice study [45], and our patients had significant differences in age between groups; thus, age was adjusted on the analysis of the cytokine expression in the urine specimens, and the results revealed that IL-1β was significantly lower in KD patients, compared with the UTI group, and there was no significant difference between pre- and post-IVIG treatment.

The limitations of this study include that age ranges were different in the three groups; nevertheless, age was adjusted in statistical analysis to avoid the confounding effect. Moreover, this study included the fever patients in the control group since it is not easy for clinicians to differentiate between infants with fever caused by viral or bacterial infection and infants with fever caused by KD. Additionally, UTI was another control group, as pyuria may be found in KD patients. The non-febrile patients’ urinary cytokines were low or almost undetectable in the other study, thus it was not collected in this study. [11]. Additionally, plasma cytokine data were not collected to correlate with urinary cytokines. A study that includes age-matched febrile/UTI controls and collection of blood and urine at the same time will be conducted later to better clarify the expression of urinary cytokines and clinical parameters that are unique to KD. Multiple cytokines are involved in the inflammatory process of KD, having overlapping, antagonistic, and synergetic effects on many cell types and upregulating and downregulating the production of other cytokines and inflammatory markers [9,10,40]. Furthermore, to evaluate a full profile of urinary T_H_1, T_H_2, T_H_17, Treg cytokines, proinflammatory, and anti-inflammatory cytokines would be to obtain a more complete picture of the evolving inflammatory process in the KD disease process before and after therapy. The multianalyte assay used in this study would be able to generate full urinary cytokine profiles for each pediatric patient while requiring less sample, time, expense, and labor than ELISA assays. Importantly, obtaining urine from pediatric patients is less invasive. The suitability of this multianalyte assay has been assessed in blood, urine, and saliva [14,15]. This profile of markers has not been used in the urine of KD patients before, and it helps to unravel the profile of urinary cytokines in the pathogenesis of KD using a less invasive method.

## 5. Conclusions

This pioneering study focused on urinary cytokines in KD. The profile of markers described in this study has not been used before in evaluating the urine of KD patients, and we showed that it helps to unravel the profile of urinary cytokines in the pathogenesis of KD using a less invasive method.

## Figures and Tables

**Table 1:** Demographic and clinical data of included patients.

	FC (*n* = 42)	KD (*n* = 37)				UTI (*n* = 28)	*p* * Value
	Median (IQR)	Median (IQR)				Median (IQR)	
		All (*n* = 37)	CAL (*n* = 10)	No CAL (*n* = 27)	*p* Value		
Age (months)	40.59 (13.50, 60.25)	23.25 (8.50, 26.50)	9.00 (6.50, 23.75)	19.00 (11.00, 27.00)	0.105	3.67 (1.68, 3.90)	<0.001
Gender (F/M), *n*	21/21	11/26	0/10	11/16	0.018	8/20	0.095
Body weight (kg)	15.87 (10.63, 7.73)	12.07 (8.65, 11.45)	10.20 (7.43, 10.38)	10.20 (8.68, 11.58)	0.158	6.02 (5.07, 6.90)	<0.001
WBC (1000/μL)	10.69 (6.40, 14.40)	12.43 (9.40, 14.70)	15.05 (9.88, 19.20)	12.85 (10.55, 14.48)	0.775	14.55 (12.95, 17.70)	0.003
Hemoglobin (g/dL)	12.26 (11.60, 12.90)	11.24 (10.50, 11.95)	10.35 (9.88, 10.75)	11.70 (10.88, 12.03)	0.062	11.80 (10.10, 13.78)	0.001
Hematocrit (%)	36.36 (34.4, 38.50)	34.00 (31.80, 36.00)	31.80 (31.30, 32.65)	34.20 (32.65, 36.65)	0.169	34.31 (29.98, 36.08)	0.001
Platelet (1000/μL)	228.7 (180.0, 279.0)	410.8 (299.0, 485.0)	463.0 (356.0, 597.5)	353.0 (285.8, 495.0)	0.371	380.7 (323.0, 450.5)	<0.001
BUN (mg/dL)	8.56 (5.50, 11.00)	7.22 (4.00, 9.00)	5.50 (4.25, 9.00)	7.00 (4.00, 9.00)	0.621	7.65 (6.00, 8.25)	0.261
Cr (mg/dL)	0.39 (0.34, 0.49)	0.32 (0.22, 0.37)	0.24 (0.21, 0.32)	0.30 (0.26, 0.41)	0.106	0.29 (0.22, 0.31)	0.007
CRP (mg/L)	32.58 (4.48, 46.43)	65.78 (22.45, 100.95)	87.10 (61.03, 128.65)	49.50 (16.95, 80.38)	0.044	30.53 (6.70, 48.95)	0.001
U-WBC (/uL)	2.95 (0.00, 3.00)	40.15 (0.00, 31.50)	10.50 (0.75, 42.75)	4.50 (0.00, 27.75)	0.223	98.77 (10.50, 171.0)	<0.001
U-RBC (/uL)	2.79 (0.00, 3.00)	5.23 (0.00, 6.00)	3.00 (0.75, 6.00)	1.50 (0.00, 6.75)	0.460	41.68 (6.75, 53.25)	<0.001
U-Cr (mg/dL)	54.34 (29.40, 70.05)	24.96 (9.09, 29.60)	13.09 (3.11, 26.54)	21.67 (11.23, 36.89)	0.158	13.29 (6.22, 15.13)	<0.001

Kruskal–Wallis test was used for comparing fever-control (FC), all KD, and UTI groups with *p* * values presented. Mann–Whitney *U* test was used for comparison of coronary artery lesion (CAL) and no CAL groups, with *p* value presented. The chi-squared or Fisher’s exact test was used for the analysis of categorical variables and expressed as numbers. Continuous variables are expressed as median (IQR). BUN: blood urea nitrogen, CRP: C-reactive protein, U-WBC: urine white count cells, RBC: red blood cells, Cr: creatinine.

**Table 2:** Differences in urinary cytokines between fever-control, Kawasaki disease, and urinary tract infection groups.

Cytokines (pg/mg)	FC (*n*= 42)	KD (*n*= 37)	UTI (*n*= 28)
EGF	38,043 (23,587, 59,132) ***	109,703 (68,870, 191,054)	144,115 (84,484, 269,474)
IFN-γ	0.65 (0.28, 1.00) ***	1.90 (0.75, 4.35)	3.59 (2.59, 10.74) *
IL-1β	0.21 (0.1, 0.43) ***	0.72 (0.44, 2.08)	43.32 (6.20, 465.3) ***
IL-2	0.33 (0.16, 0.51) **	0.88 (0.42, 1.66)	0.69 (0.34, 1.01)
IL-4	2.52 (1.15, 4.67) ***	9.32 (6.42, 16.16)	9.04 (5.28, 22.44)
IL-5	0.48 (0.29, 0.66) ***	2.00 (1.41, 3.01)	1.50 (0.65, 2.25)
IL-6	1.32 (0.55, 4.68) **	81.71 (10.54, 219.8)	5.28 (2.14, 12.36) ***
IL-8	5.34 (1.42, 17.93) **	36.81 (6.95, 115.4)	1713 (180.7, 8324) ***
IL-9	2.45 (1.30, 3.80) ***	8.98 (5.27, 14.20)	5.85 (3.73, 10.49)
IL-10	1.7 (0.85, 2.89) ***	10.17 (7.06, 27.18)	6.00 (2.80, 16.35)
IL-13	0.40 (0.25, 0.70) ***	2.10 (1.20, 6.10)	1.86 (1.22, 2.98)
IL-17A	0.70 (0.39, 0.99) **	1.34 (0.78, 3.24)	4.88 (2.82, 18.69) ***
IL-33	18.43 (7.32, 38.29) **	354 (229, 668.8)	54.50 (17.85, 165.7) ***
IP-10	279.4 (41.88, 920) *	1259 (264.7, 5723)	1312 (107.1, 5115)
MCP-1	849.1 (355.8, 1926)	4836 (2440, 14,231)	2441 (667.3, 3469) **
MIP-1β	6.90 (2.45, 13.20) ***	87.66 (27.70, 126.3)	31.74 (12.81, 50.46) **
TNF-α	0.50 (0.26, 0.92) ***	12.47 (2.05, 68.96)	1.16 (0.73, 2.89) ***
VEGF	45.58 (2.33, 103.8) ***	258.6 (132.1, 498.4)	145.4 (64.00, 374.4)

Data are presented as median (IQR), by Kruskal–Wallis test. Asterisk represents significant difference between Kawasaki disease (KD) vs. fever-control (FC) or KD vs. urinary tract infection (UTI) groups, * for *p* < 0.05, ** for *p* < 0.01 and *** for *p* < 0.001.

**Table 3:** The levels of urinary cytokines in fever-control, Kawasaki disease, and UTI groups before and after logistic regression analysis.

Cytokines (pg/mg)	Groups	Initial Analysis			Logistic Regression (Adjusting for Age)			
		Median	IQR	*P*	OR	95% CI		*p* *
						Lower	Upper	
EGF	FC	38,043	(23,587, 59,132)	<0.001	0.997	0.995	0.998	<0.001
	KD	109,703	(68,870, 191,054)					
	UTI	144,115	(84,484, 269,474)		1.000	1.000	1.000	0.827
IFN-γ	FC	0.65	(0.28, 1.00)	<0.001	0.216	0.097	0.479	<0.001
	KD	1.90	(0.75, 4.35)					
	UTI	3.59	(2.59, 10.74)		1.008	0.927	1.096	0.853
IL-1β	FC	0.21	(0.10, 0.43)	<0.001	0.666	0.385	1.154	0.148
	KD	0.72	(0.44, 2.08)					
	UTI	43.32	(6.20, 465.3)		1.282	1.008	1.631	0.043
IL-8	FC	5.34	(1.42, 17.93)	<0.001	0.978	0.963	0.994	0.006
	KD	36.81	(6.95, 115.4)					
	UTI	1713	(180.7, 8324)		1.007	0.999	1.014	0.079
IL-13	FC	0.40	(0.25, 0.70)	<0.001	0.258	0.122	0.543	<0.001
	KD	2.10	(1.20, 6.10)					
	UTI	1.86	(1.22, 2.98)		0.904	0.783	1.043	0.165
IL-17A	FC	0.70	(0.39, 0.99)	<0.001	0.286	0.136	0.602	0.001
	KD	1.34	(0.78, 3.24)					
	UTI	4.88	(2.82, 18.69)		1.101	0.951	1.274	0.198

The *p* values represent the differences in levels of cytokines between the three groups by Kruskal–Wallis test. Additionally, *p* * values present the differences of levels of cytokines between the fever-control (FC) vs. KD, or UTI vs. KD groups by the logistic regression analysis after adjusting age. OR, odds ratio, CI: confidence interval.

## Data Availability

The data presented in this study are available on request from the corresponding author. The data are not publicly available due to ethical considerations.
